# Antitumour Activity of the Ribonuclease Binase from *Bacillus pumilus* in the RLS_40_ Tumour Model Is Associated with the Reorganisation of the miRNA Network and Reversion of Cancer-Related Cascades to Normal Functioning

**DOI:** 10.3390/biom10111509

**Published:** 2020-11-02

**Authors:** Islam Saber Ead Mohamed, Aleksandra V. Sen’kova, Alsu I. Nadyrova, Innokenty A. Savin, Andrey V. Markov, Vladimir A. Mitkevich, Aleksander A. Makarov, Olga N. Ilinskaya, Nadezhda L. Mironova, Marina A. Zenkova

**Affiliations:** 1 Institute of Chemical Biology and Fundamental Medicine, Siberian Branch of the Russian Academy of Sciences, Novosibirsk 630090, Russia; sabermohamedm28@gmail.com (I.S.E.M.); alsenko@mail.ru (A.V.S.); kesha_savin@mail.ru (I.A.S.); andmrkv@gmail.com (A.V.M.); marzen@niboch.nsc.ru (M.A.Z.); 2 Department of Natural Sciences, Novosibirsk State University, Novosibirsk 630090, Russia; 3 Institute of Fundamental Medicine and Biology, Kazan Federal University, Kazan 420008, Russia; alsu.nadyrova@yandex.ru (A.I.N.); ilinskaya_kfu@mail.ru (O.N.I.); 4 Engelhardt Institute of Molecular Biology, Russian Academy of Sciences, Moscow 119991, Russia; mitkevich@gmail.com (V.A.M.); aamakarov@eimb.ru (A.A.M.)

**Keywords:** RNases, binase, cytotoxicity, miRNAs, antitumour activity, tumour models

## Abstract

The important role of miRNA in cell proliferation and differentiation has raised interest in exogenous ribonucleases (RNases) as tools to control tumour-associated intracellular and extracellular miRNAs. In this work, we evaluated the effects of the RNase binase from *Bacillus pumilus* on small non-coding regulatory RNAs in the context of mouse RLS_40_ lymphosarcoma inhibition. In vitro binase exhibited cytotoxicity towards RLS_40_ cells via apoptosis induction through caspase-3/caspase-7 activation and decreased the levels of miR-21a, let-7g, miR-31 and miR-155. Intraperitoneal injections of binase in RLS_40_-bearing mice resulted in the retardation of primary tumour growth by up to 60% and inhibition of metastasis in the liver by up to 86%, with a decrease in reactive inflammatory infiltration and mitosis in tumour tissue. In the blood serum of binase-treated mice, decreases in the levels of most studied miRNAs were observed, excluding let-7g, while in tumour tissue, the levels of oncomirs miR-21, miR-10b, miR-31 and miR-155, and the oncosuppressor let-7g, were upregulated. Analysis of binase-susceptible miRNAs and their regulatory networks showed that the main modulated events were transcription and translation control, the cell cycle, cell proliferation, adhesion and invasion, apoptosis and autophagy, as well as some other tumour-related cascades, with an impact on the observed antitumour effects.

## 1. Introduction

The microRNAs (miRNAs) discovered in animals and plants [1] are non-coding RNAs with a length of 19–25 nucleotides [2,3] that regulate gene expression at the post-transcriptional level via specific complementary sites of target mRNAs, causing translational repression or degradation [4]. miRNAs have been found to be significant modulators of a variety of cellular processes [5], including angiogenesis [6], apoptosis [7], the cell cycle [8], proliferation [9] and telomerase activity [10]. They are thought to play a key role in disease development, particularly in oncological diseases [11]. An altered miRNA expression profile has been related to the development of different tumours [11].

Endogenous ribonucleases (RNases) participate in the maintenance of normal homeostasis of cellular RNAs. They regulate miRNA biogenesis, RNA decay, cleave aberrant RNAs and control the turnover of transcripts [12]. Taking into account the important role of RNases inside the cell, it can be suggested that exogenous RNases can take on the role of endogenous RNases when they are insufficiently functioning. These findings direct attention to exogenous RNases as tools to compensate for the malfunction of endogenous ones.

Currently, the most well-studied exogenous RNases with established antitumour activity are fungal RNases (α-sarcin, mitogillin and restrictocin) [13,14,15], BS-RNase from bull testes [16], amphibian RNase onconase from the oocytes of *Rana pipiens* (onconase) [17], bovine pancreatic RNase A [18,19] from the RNase A superfamily, the microbial RNase barnase from *Bacillus amyloliquefaciens* [20] and binase from *Bacillus pumilis* [21,22,23,24,25,26,27,28,29,30,31] belonging to the RNase T1 superfamily [24].

The ability of binase to retard primary tumour growth and inhibit metastasis formation has been demonstrated in several experimental mouse tumour models [24,28,32,33]. The antitumour activity of binase was shown to be dependent on the expression of some oncogenes, i.e., *KIT*, *AML1-ETO* and *FLT*3 [34,35], and the cleavage of certain intracellular RNAs, initiation of the intrinsic apoptotic pathway through changes in mitochondrial potential and the extrinsic apoptotic pathway via the increased expression of some pro-apoptotic genes, including cell death ligand TNF-α and the activation of initiator caspases [23]. Nevertheless, the impact of the RNase activity of binase and the involvement of its RNA targets in this antitumour effect have not yet been studied.

Here, we assessed the effect of binase on small non-coding regulatory RNAs in the context of inhibiting tumour progression. The antitumour potential of binase, which was evaluated in vitro and in vivo against mouse RLS_40_ lymphosarcoma, was compared with alterations in the miRNA profile caused by binase. Bioinformatic analysis of the possible pathways regulated by the miRNA affected by binase in cells and tumour tissue showed that binase altered the levels of particular miRNAs and modulated the tumour microenvironment, with an influence on the differentiation of tumour microenvironment-related cells.

## 2. Materials and Methods

### 2.1. Binase Preparation and Purification

Binase (12.3 kDa) was isolated as a homogenous protein from the culture medium of *Escherichia coli* BL21 cells containing the plasmid pGEMGX1/ent/Bi. Binase was purified as described earlier [36]. The endotoxin content in the binase preparations, as determined by the Limulus amoebocyte lysate test (LAL) (Charles River Endosafe Ltd., Charleston, SC, USA), was less than 5 EU/mg. The catalytic activity of binase was studied using poly(I) as the substrate [36].

### 2.2. Cell Culture

Modified RLS_40_ cells were obtained from the cell collection of the Institute of Chemical Biology and Fundamental Medicine, Siberian Branch of the Russian Academy of Sciences (SB RAS) (Novosibirsk, Russia). RLS_40_ cells were cultured in complete IMDM medium (10% foetal bovine serum (FBS), 100 units/mL penicillin, 100 μg/mL streptomycin and 2 mM glutamine) under standard conditions (at 37 °C in a humidified atmosphere with 5% CO_2_).

### 2.3. Mice

Male 10- to 14-week-old CBA/LacSto (hereinafter, CBA) mice were provided by the vivarium of the Institute of Chemical Biology and Fundamental Medicine SB RAS, Novosibirsk, Russia. The mice were housed in plastic cages (10 animals per cage) under normal daylight conditions. Water and food were provided ad libitum.

All animal procedures were performed in strict accordance with the approved protocol and recommendations for the proper use and care of laboratory animals (ECC Directive 2010/63/EU). The experimental protocols were approved by the Committee on the Ethics of Animal Experiments with the Institute of Cytology and Genetics SB RAS (ethical approval number 50 from 23/5/2019) and all efforts were made to minimise suffering.

### 2.4. Viability of RLS_40_ Cells

Cells were seeded onto 96-well plates (0.4 × 10^5^ cells/well) in serum-free IMDM medium and binase was added at concentrations ranging from 50–700 µg/mL. Plates were incubated under standard conditions for 48 h. The viability was assessed using a WST-1-based test (Sigma-Aldrich, Darmstadt, Germany). WST-1 solution (Sigma-Aldrich, Darmstadt, Germany) was added according to the manufacturer’s protocol and the cells were incubated for another 3–4 h under the same conditions. The optical density of the resulting solution (A) was measured at wavelengths of 450 and 620 nm on a multichannel Multiscan RC spectrophotometer (Labsystems, Helsinki, Finland). The activity of the mitochondrial dehydrogenases was calculated as the difference in absorbance ΔA = (A_450_ − A_620_). The respiratory activity of untreated cells was taken to be 100%. All reported values are the means of three independent measurements  ±  SD.

### 2.5. Treatment of RLS_40_ Cells with Binase for miRNA Analysis

RLS_40_ cells were seeded in triplicate on 12-well plates at a density of 1.5 × 10^6^ cells/well in antibiotic-free IMDM supplemented with 10% FBS (Sigma-Aldrich, Darmstadt, Germany). Binase was added at the concentrations of 5 and 10 µg/mL and the cells were incubated under standard conditions for 24 h, followed by RNA isolation.

### 2.6. Annexin V FITC/PI Apoptosis Detection

RLS_40_ cells were seeded in 24-well plates at a density of 1.5 × 10^6^ cells per well and incubated in antibiotic-free IMDM supplemented with 10% FBS (Sigma-Aldrich, St Louis, MO, USA) in the presence of binase (500 µg/mL) for 48 h under standard conditions. The medium was discarded and the cells were gently suspended in a binding buffer at a concentration of 10^6^ cells/mL. Then, the cells were stained with fluorescein isothiocyanate (FITC)-conjugated Annexin V and PI (Annexin V-FITC Apoptosis Detection Kit, Millipore, Bedford, MA, USA) for 15 min in the dark and immediately analysed using a NovoCyte flow cytometer (ACEA Biosciences Inc., San Diego, CA, USA).

### 2.7. Determination of Caspase-3/-7 Activity and Caspase-3/-7-Positive Cells

The caspase-3/-7 activity in RLS_40_ cells was assessed using a Caspase-Glo^®^ 3/7 Assay kit (Promega, Madison, WI, USA) according to the manufacturer’s instructions. Briefly, the cells were seeded in white-walled 96-well plates at a density of 0.4 × 10^5^ cells/well and incubated in serum- and antibiotic-free IMDM in the presence of binase (500 µg/mL) for 48 h under standard conditions. Further procedures were performed according to the manufacturer’s protocol. Luminescence was detected using a CLARIOstar plate reader (BMG Labtech, Ortenberg, Germany).

The plates were prepared as described in previous paragraph. Further procedures were performed according to the manufacturer’s protocol. The number of caspase-3/-7-positive cells was assessed using CellEvent^®^ Caspase-3/7 Green Flow Cytometry Assay Kit (Life Technologies, Eugene, OR, USA) analysed using a NovoCyte flow cytometer (ACEA Biosciences Inc., San Diego, CA, USA).

### 2.8. Tumour Transplantation and the Design of the Animal Experiments

Solid tumour RLS_40_ cells were generated via the intramuscular (i.m.) injection of RLS_40_ cells (10^7^ cells/mL) suspended in 0.1 mL of saline buffer into the right thigh of CBA mice. On day 4 after the tumour implantation, mice with RLS_40_ were divided into three groups (*n* = 20 per group): (1) the control group received an intraperitoneal injection (i.p.) of saline buffer (0.1 mL), and (2) and (3) received i.p. binase at the doses of 0.5 mg/kg and 1 mg/kg three times per week, respectively. The total number of injections was seven.

The tumour sizes were monitored every other day using calliper measurements in three perpendicular dimensions. Tumour volumes were calculated as V = length × width × height × (π/6). Mice were sacrificed 1 h after the final injection of binase (on day 18 after the tumour implantation) via cervical dislocation. For the analysis of the miRNA expression profile, tumour and blood samples were taken. For histological and morphometric analysis, liver, kidney, spleen, thymus and tumour samples were collected.

### 2.9. Sample Processing and RNA Extraction

The isolation of total RNA from RLS_40_ was carried out using a TRIzol reagent (Sigma-Aldrich, Darmstadt, Germany) according to the manufacturer’s protocol. Tumour pieces from the mice of the control and experimental groups were homogenised. Blood serum was prepared from the whole blood of mice via clot formation at 37 °C for 30 min and at 4 °C overnight, followed by clot discarding and centrifugation (4000 rpm, 4 °C, 20 min). Serum samples were pooled according to groups. Total RNA was extracted from the serum samples, RLS_40_ cells and homogenates of the tumour tissue immediately using a TRIzol reagent (Sigma-Aldrich, Darmstadt, Germany) according to the manufacturer’s protocol.

RNA concentration was measured via absorbance at 260 and 280 nm using a Bio Mate 3 (Thermo Electron Corporation, Waltham, MA, USA) spectrophotometer and Qubit (Invitrogen, Carlsbad, CA, USA). The total RNA integrity and quantity were checked using Agilent 2100 Bioanalyzer (Agilent Technologies, Santa Clara, CA, USA).

### 2.10. qPCR

The expression of miRNA in RLS_40_ cells was analysed using stemloop PCR technology [37,38]. cDNA synthesis was performed using M-MuLV reverse transcriptase (Biolabmix, Novosibirsk, Russia). The reverse transcription reaction was performed in a total volume of 40 µL containing 5 µg of total RNA, 5× RT buffer mix, 100 units of M-MuLV-RH reverse transcriptase and 0.05 µM of miRNA-specific stem-loop primers (Table S1). The RT reaction involved the following: 16 °C, 30 min; 30 °C, 30 s, 40 cycles; 42 °C, 30 s; a final reverse transcriptase inactivation at 85 °C for 5 min.

PCR was performed in a total volume of 20 µL using 2× BioMaster HS-qPCR SYBR Blue (Biolabmix, Novosibirsk, Russia) and 0.05 µM of forward miRNA-specific primers and universal reverse primer (Table S2). The PCR conditions were as follows: 95 °C, 5 min; 95 °C, 15 s, 40 cycles; 58 °C, 15 s; 72 °C, 30 s; 75 °C, 15 s; followed by a melting point determination. The obtained PCR data were analysed using standard Bio-Rad iQ5 v.2.0 software (Bio-Rad Laboratories, Hercules, CA, USA). For each sample, the threshold cycle (Ct) was determined. Quantitative assessment of the level of transcripts representation and relative miRNA expression in tumour cells and tumour tissue was performed by comparing the Ct values for miRNA and the reference U6. The concentration of serum-derived miRNAs was normalised to the serum volume.

### 2.11. Histology and Morphometry

Tissue samples (liver, kidney, spleen, thymus and tumour) were fixed in 10% neutral buffered formalin (BioVitrum, St. Petersburg, Russia), dehydrated in ascending ethanols and xylols and embedded in HISTOMIX paraffin (BioVitrum, St. Petersburg, Russia). Paraffin sections (5 μm) were sliced on a Microm HM 355S microtome (Thermo Fisher Scientific, Waltham, MA, USA) and stained with haematoxylin and eosin. For the immunohistochemical study, the tumour sections (3–4 μm) were deparaffinised and rehydrated. Antigen retrieval was carried out after exposure in a microwave oven at 700 W. The samples were treated with anti-PCNA (2139510, Sony Biotechnology, San Jose, CA, USA), anti-caspase-3-specific (ab2302, Abcam, Cambridge, MA, USA) and anti-caspase-7-specific (MAB823, R&D Systems, Minneapolis, MN, USA) monoclonal antibodies according to the manufacturer’s protocol. Then, the sections were incubated with secondary horseradish peroxidase (HPR)-conjugated antibodies (Spring Bioscience detection system, Spring Bioscience, Pleasanton, CA, USA), exposed to a 3,3′-diaminobenzidine (DAB) substrate and stained with Mayer’s haematoxylin. All images were examined and scanned using an Axiostar Plus microscope equipped with an Axiocam MRc5 digital camera (Zeiss, Oberkochen, Germany) at magnifications of ×100 and ×400.

The percentages of the internal metastases areas were determined relative to the total area of the sections using Adobe Photoshop software. The inhibition of metastasis development was assessed using the metastasis inhibition index (MII), calculated as MII = ((mean metastasis area_control_ − mean metastasis area_experiment_)/mean metastasis area_control_) × 100%.

Morphometric analysis of the tumour, spleen and thymus sections was performed via point counting using a morphometric grid, which consisted of 100 testing points in a testing area equal to 3.2 × 10^6^ μm^2^. Morphometric analysis of the tumour tissue included the evaluation of the volume densities (Vv (%)) of the unchanged tumour tissue, lymphoid infiltration, necrosis and numerical densities (Nv) of mitoses and PCNA-positive cells in the tumour tissue. Morphometric analysis of the spleen included the evaluation of the volume densities (Vv (%)) of the red pulp, the white pulp and the diameter of the lymphoid follicles (µm). Morphometric analysis of the thymus included the evaluation of the volume densities (Vv (%)) of the cortex and medulla with a subsequent calculation of the cortex/medulla index. The histological study of the liver and kidney included the evaluation of destructive changes (dystrophy and necrosis) of hepatocytes and epitheliocytes in the proximal tubules.

The volume density (Vv (%)) of a studied histological structure represented the volume of the tissue fraction occupied by this compartment and determined from testing points lying over this structure and calculated using the following formula: Vv = (P_structure_/P_test_) × 100%, where P_structure_ denotes the number of points over the structure and P_test_ denotes the total number of test points, which was 100 in this case. The numerical density (Nv) of the studied histological structure indicated the number of particles in the unit tissue volume evaluated as a number of particles in the square unit, which was 3.2 × 10^6^ μm^2^ in this case.

Each studied group included 20 mice and 10 random fields were studied in each organ specimen, forming a total of 200 testing fields for each group of mice.

### 2.12. Blood Biochemistry

Biochemical parameters of the blood serum of the experimental animals were estimated using an HTI BioChem FC-200 Auto Chemistry Analyzer (High Technology Inc., North Attleboro, MA, USA). The serum levels of alanine aminotransferase (ALT), aspartate aminotransferase (AST), alkaline phosphatase (ALK), total protein, creatinine and blood urea nitrogen (BUN) (HT-A206-120, HT-A109-120, HT-A205-120, HTT251-125, HT-C225-250, and HT-B253-150, High Technology Inc., North Attleboro, MA, USA) were measured.

### 2.13. miRNA Target Prediction and Functional Analysis

The target genes of binase-susceptible miRNAs were predicted using the CyTargetLinker v. 4.1.0 plugin [39] based on miRNA-target interactions deposited in the experimentally verified miRTarBase v. 8.0 database (*Mus musculus*) [40]. The obtained regulatory interaction network was visualised using Cytoscape v. 3.7.2 (The Cytoscape Consortium, San Diego, CA, USA). Functional analysis of the revealed target genes was carried out using the ClueGO v. 2.5.4 plugin [41] in Cytoscape. Genes were mapped on the Gene Ontology (Biological Processes) (The Gene Ontology Consortium, USA), Kyoto Encyclopedia of Genes and Genomes (KEGG) (Kyoto University and University of Tokyo, Japan), REACTOME (The Reactome group, USA and UK) and WikiPathways (The WikiPathway Team, USA, The Netherlands, Austria, Brasil) databases. Term enrichment was tested with a two-sided hypergeometric test that was corrected using the Bonferroni method. Only terms with *p* ≤ 0.05 were included in the analysis. Functional grouping and linking of the enriched terms were performed with kappa statistics (kappa score = 0.4).

The key common genes were then analysed from the point of view of their participation in cancer-related pathways and events using the GeneCards [42] and KEGG databases [43].

### 2.14. Statistics

All experiments were reproduced in triplicate. Data were statistically processed using Student’s *t*-test (two-tailed, unpaired) or one-way analysis of variance (ANOVA). Post hoc testing was completed using a post hoc Tukey’s test; *p* < 0.05 was considered to be statistically significant. The statistical package STATISTICA version 10.0 (StatSoft, Tulsa, OK, USA) was used for analysis.

## 3. Results

### 3.1. The Choice of the Tumour Model

The first study of the ability of binase to retard primary tumour growth and metastasis development was performed using three tumour models of different histological types with strong relevance to human tumours: Lewis lung carcinoma (LLC) with an epithelial origin is related to human non-small cell lung cancer [44]; mouse RLS_40_ lymphosarcoma is derived from hematopoietic tissue and displays a multidrug resistance phenotype related to human diffuse large B-cell lymphoma; melanoma B-16 arose from neuroectodermal tissue and is related to human metastatic melanoma [45]. LLC and RLS_40_ are able to form primary solid tumour nodes after intramuscular implantation, and these tumours metastasise into the lungs and liver, respectively; after intravenous implantation, melanoma B16 can only form metastases in the lungs.

Previous data showed that intraperitoneal injections of binase (dose range 1–5 mg/kg) resulted in the retardation of primary tumour growth by up to 45% in the LLC and RLS_40_ models and inhibited metastasis development by up to 50% in the case of LLC and RLS_40_ and up to 70% in B16 melanoma [28]. In this work, to study the antitumour potential of binase in detail, we chose RLS_40_ lymphosarcoma, taking into account several specific characteristics of this tumour. First of all, RLS_40_ forms primary tumour nodes and metastasises to the liver. Since binase, like any xenobiotic, will be metabolised in the liver, this model allowed for tracking a triple burden on the liver, i.e., from the tumour, from metastases, and from metabolism. Second, RLS_40_ lymphosarcoma is a tumour with a multiple-drug-resistant phenotype that has a disrupted system of ABC transporters. This system allows for chemotherapeutics to be pumped out of cells. Binase, being a protein, allowed us to bypass this system and excluded it from the confrontation with chemotherapeutic therapy. A disrupted ABC transporter system can cause an alteration in the mechanism of the antitumour activity of binase.

### 3.2. The Effect of Binase on the Proliferation of RLS_40_ Cells and Apoptosis Induction

**Figure 1:**
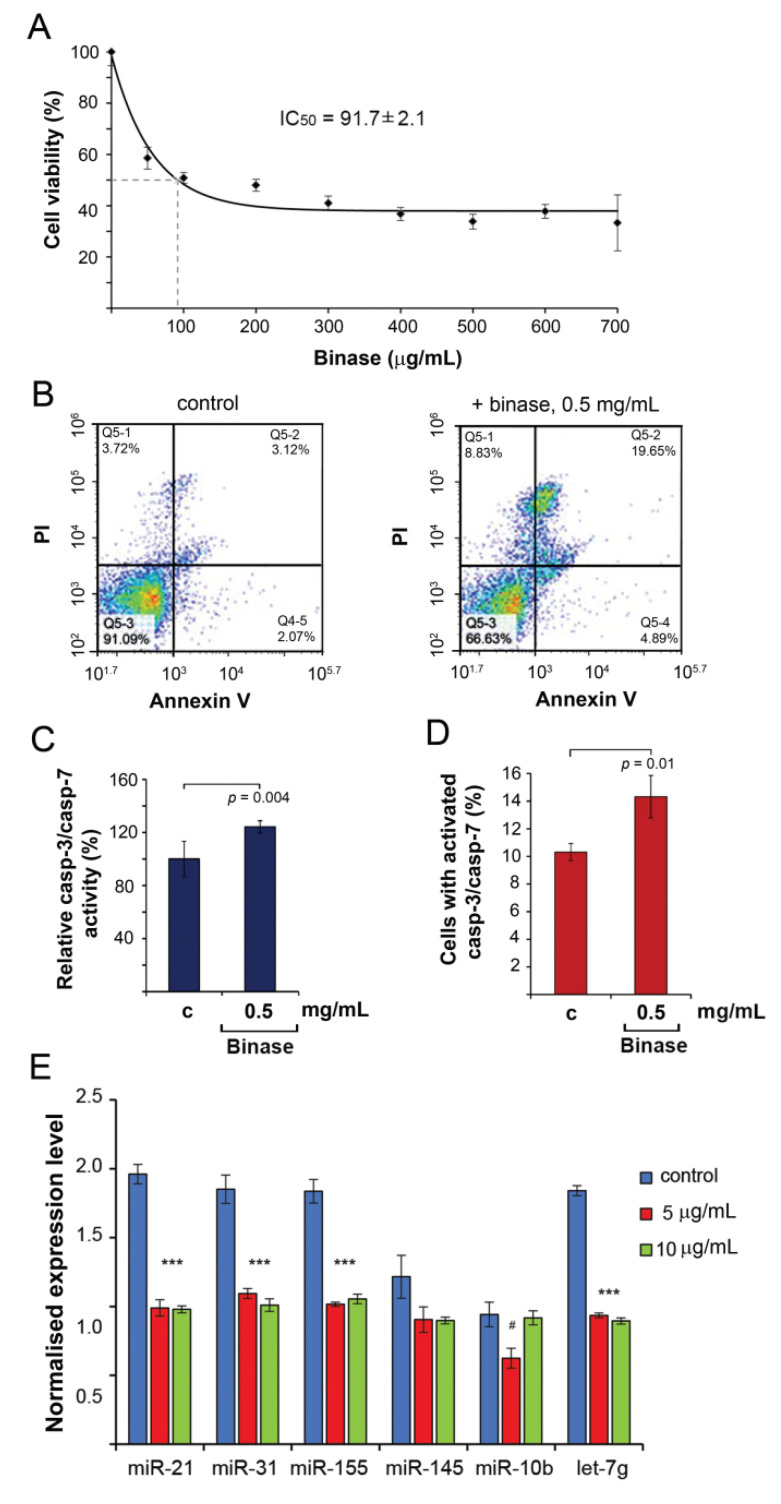
The effect of binase on mouse RLS_40_ cells. (**A**) Viability of RLS_40_ cells in the presence of binase. RLS_40_ cells were incubated with binase for 48 h. The number of living cells in the control was set to be 100% (the cells were incubated in the absence of binase). The values are represented as mean ± SE. (**B**) Induction of apoptosis in RLS_40_ cells by binase. Cells were incubated in the presence of binase (0.5 mg/mL) for 48 h. Untreated cells were used as the control. The cells were stained by Annexin V FITC/PI and analysed using flow cytometry. The percentage of live (lower-left quadrant), early apoptotic (lower-right quadrant), late apoptotic (upper-right quadrant) and necrotic (upper-left quadrant) cells are shown. (**C**) Activation of caspase-3/-7 in RLS_40_ cells treated with binase. The cells were treated with binase and the caspase-3/-7 activity was measured using a Caspase-Glo^®^ 3/7 Assay kit (Promega, Madison, WI, USA). (**D**) The effect of binase on the cell RLS_40_ population with activated caspase-3/-7. Cells were treated with binase and the number of cells with activated caspases was measured using the CellEvent^®^ Caspase-3/7 Green Assay (Life Technologies, Eugene, OR, USA). (**E**) Alteration of the miRNA expression after the RLS_40_ cell treatment with binase (5 and 10 μg/mL). Data of stem-loop RT-qPCR. miRNA expression levels were normalised to U6 snRNA. *** *p* < 0.05; ^#^ statistically insignificant. The data are presented as the mean of three independent experiments with triplicate samples ± SD. Data were statistically processed using Student’s *t*-tests (**A**–**D**) and one-way ANOVA (**E**) with the Tukey’s post hoc test; *p* < 0.05 was considered to be statistically significant.

RLS_40_ cells were treated with binase at concentrations ranging from 50 to 700 μg/mL (Figure 1A). From the presented data, it can be seen that at the 100 µg/mL concentration of binase, the viability of the RLS_40_ cells decreased by 50%, and in the range of concentrations of 400–700 µg/mL, the cell survival rate was about 30% (Figure 1A). Based on the WST-1 test data, the IC_50_ value of binase relative to the RLS_40_ cell line was 91 µg/mL.

We then measured the apoptosis induction in the RLS_40_ cells incubated with binase. In the case of apoptosis, we used a concentration of binase corresponding to 5 × IC_50_ to reach a pronounced and well-documented effect. Indeed, the cytotoxic effect of binase on the RLS_40_ cells was manifested by apoptosis induction (Figure 1B). The percentage of apoptotic cells in the population treated with binase (0.5 mg/mL, 48 h) increased by a factor of 6.3 in comparison with untreated cells and reached approximately 20% (Figure 1B, right panel); these were late apoptotic cells (Annexin V FITC+/PI+, right-upper quadrant, Figure 1B). Besides this, the percentage of early apoptotic and necrotic cells was only slightly increased, as the observed increase of these cell populations did not exceed 4 and 5%, respectively (Figure 1B).

Binase-induced apoptosis was accompanied by the activation of key executioner caspase-3 and caspase-7 in the RLS_40_ cells (Figure 1C). Moreover, we found that the cell treatment with binase resulted in the accumulation of active caspase-3/-7-expressing cells (Figure 1D). Thus, our findings demonstrated that binase inhibited the viability of RLS_40_ lymphosarcoma cells by triggering caspase-dependent apoptosis.

### 3.3. The Effect of Binase on miRNA Expression Levels in RLS_40_ Cells

The effect of binase on a number of miRNAs in RLS_40_ cells was investigated. The miRNA panel included miR-21a, miR-10b, miR-14a5, miR-31, let-7g and miR-155 (Table 1). These miRNAs are in the list of top 50 miRNAs according to the algorithm of miRNA selection, which consisted of the profiling of miRNA expression in blood serum and tumour tissue of LLC-bearing mice treated with RNase A and additional sorting of the identified miRNAs by abundance score (reads per kilobyte per million (RPKM) values) and by fold change in descending order [46]. Since miRNAs are tissue-specific, and LLC and RLS_40_ have epithelial and hematopoietic origin, respectively, we chose six miRNAs to provide some overlap between the miRNA profiles of these different tissues in case some of the miRNAs were not expressed at the proper level in the RLS_40_ cells. Moreover, the oncomirs miR-21a and miR-155 have been shown to be frequently overexpressed in diffuse large B-cell lymphoma [47,48], to which mouse RLS_40_ lymphosarcoma is related.

Using stem-loop RT-qPCR, it was shown that all the chosen miRNAs were noticeably expressed in RLS_40_ cells (Table 1; see expression levels of the miRNAs in RLS_40_). The most expressed miRNAs were miR-155, let-7g and miR-21a, with expression levels of 1.9, 1.8 and 1.7, respectively (Table 1), normalised to the level of the small nuclear RNA U6. miR-10b and miR-145a were expressed at a moderate level with expression levels 1.5 and 1.4, and miR-31 was expressed at the lowest level of 0.85 (Table 1).

Obviously, at the final stages of apoptosis, which was achieved after the exposure of RLS_40_ cells to binase at a concentration of 5 × IC_50_, we could not find differences in the miRNA expression. This was why for miRNA profiling, the RLS_40_ cells were incubated for 48 h at binase concentrations of 5 and 10 µg/mL. Choosing concentrations of binase that were much lower than the IC_50_ permitted us to analyse the initial events preceding apoptosis at the level of cellular regulatory RNAs. The data from stem-loop RT-qPCR showed that treatment of RLS_40_ cells with binase at a concentration of 5 μg/mL resulted in a 1.6- to 2-fold decrease in the expression levels of miR-21a, let-7g, miR-31 and miR-155 (Table 1 and Figure 1E); a further increase in the binase concentration did not potentiate this effect. The levels of miR-10b and miR-145a did not change after the binase treatment (Figure 1E).

Binase is a guanine-specific ribonuclease, thus we tried to juxtapose the number of guanine residues in miRNAs and the observed effects. Indeed, it turned out that the level of miRNAs that contained 5–7 guanine residues whose phosphodiester bond was susceptible to cleavage with binase were strongly decreased, while the levels of miR-145a and miR-10b, with four guanines, did not change after treatment with binase (Table 1). Thus, in vitro binase exhibited cytotoxic effects against RLS_40_ cells via apoptosis induced by intracellular RNA degradation.

### 3.4. The Effect of Binase on RLS_40_ Tumour Growth and Metastasis Development In Vivo

**Figure 2:**
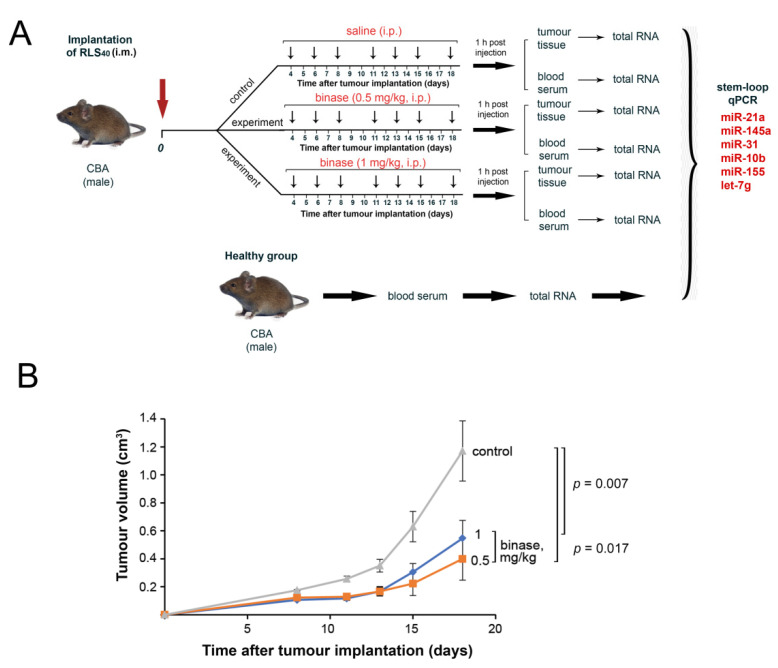
Effect of binase on the RLS_40_ tumour progression in CBA mice. (**A**) Design of the experiment. RLS_40_ cells (10^5^ cells, 0.1 mL) were intramuscularly (i.m.) implanted into CBA mice. Starting on day 4 after the tumour implantation, the animals received saline buffer or binase intraperitoneally (i.p., doses and regiment are indicated on the scheme). One hour after the last injection, tumour and blood samples were collected, the total RNA was isolated and the miRNA levels were analysed using RT-qPCR. (**B**) Dynamics of tumour growth. Mice received saline buffer (control) or binase at the doses of 0.5 and 1 mg/kg. Statistical analysis was performed using one-way ANOVA with the Tukey’s post hoc test.

The scheme of the in vivo experiment is depicted in Figure 2A. Binase significantly inhibited the growth of primary tumour nodes; at a binase dose 0.5 mg/kg, a 60% slowdown of tumour growth was observed (Figure 2B). A 2-fold increase in binase dose did not potentiate the antitumour effect, as the retardation of tumour growth was approximately the same without a statistically significant difference between the groups (Figure 2B).

**Figure 3:**
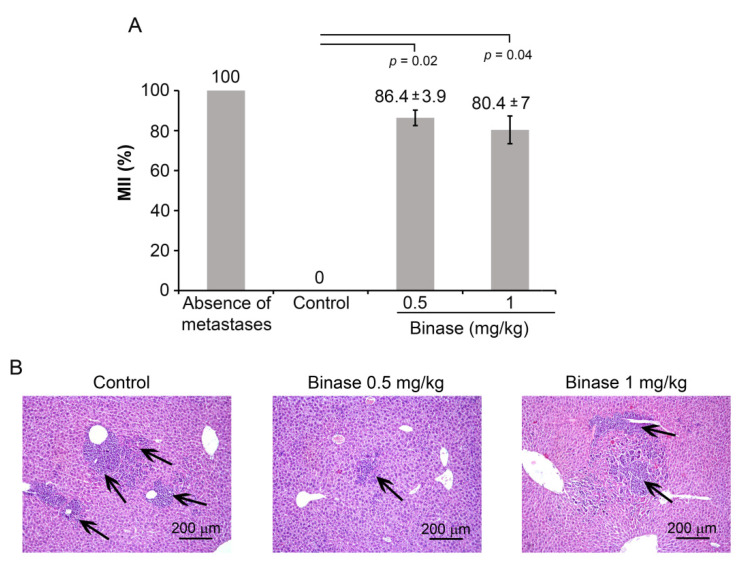
The effect of binase on the metastasis development in RLS_40_-bearing mice. (**A**) Metastasis inhibition index (MII) in the control (saline buffer) and experimental groups (binase 0.5 and 1 mg/kg). MII = ((mean metastasis area_control_ − mean metastasis area_experiment_)/mean metastasis area_control_) × 100%. The data were statistically analysed using Student’s *t*-test and are presented as mean ± SE. Statistical significance: *p* ≤ 0.05. (**B**) Representative histological images of the liver of RLS_40_-bearing mice treated with binase. Metastases are indicated by black arrows. Haematoxylin and eosin staining, original magnification ×200. Control: RLS_40_-bearing mice treated with saline buffer.

Binase administration led to the inhibition of metastasis development (Figure 3). Since most metastases in the liver were internal, we calculated the MII based on morphometric measurements of the metastasis area in relation to the total liver area. The MII in the control group was set to be 0%, and the MII corresponding to the absence of metastases was taken to be 100% (for details, see the Materials and Methods section). Binase at both doses efficiently suppressed the development of RLS_40_ liver metastases with an MII of 80–86% (Figure 3A).

Liver metastases of the RLS_40_ lymphosarcoma were represented predominately by rounded foci with unclear boundaries consisting of the large monomorphic atypical lymphoid cells, which were comparable with the cells forming the primary tumour node; the number of such foci was significantly decreased after the treatment with binase (Figure 3B).

**Figure 4:**
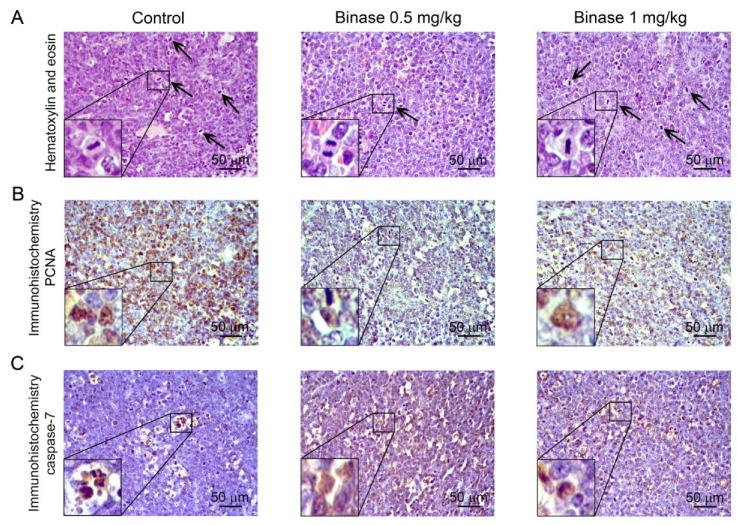
Mitoses, PCNA- and caspase-7-positive cells in RLS_40_ tumour tissue after the binase administration. Representative histological images of tumour sections. Haematoxylin and eosin staining (**A**) and immunohistochemical staining with anti-PCNA (**B**) and anti-caspase-7 (**C**) monoclonal antibodies. Mitosis events are indicated by black arrows. Magnification: ×400.

Together with a reduction in the tumour volume, binase also caused changes in the histological characteristics of the tumours. The tumours had rounded shape with clear boundaries and foci of necrosis in the central part of tumour nodes in all studied groups. The tumour tissue was represented by large monomorphic atypical lymphoid cells with a high mitotic rate and proliferative activity (Figure 4A). Morphometric analysis followed by calculations of the morphological parameters of the tumour tissue showed that in the control group, the numerical density of mitosis was 5.6 ± 0.4 per test area and the volume density of PCNA-positive tumour cells was 62.7 ± 3.8% (Table 2). In the group that received binase at a dose of 0.5 mg/kg, a 5.1-fold decrease in the numerical density of mitosis and 2.9-fold decrease in the volume density of PCNA-positive cells was observed in comparison with the control (Figure 4A,B and Table 2), whereas binase applied at a dose of 1 mg/kg did not have such pronounced effects: the number of mitoses and PCNA-positive cells decreased only by 1.8- and 1.4-fold in comparison with the control group, respectively (Figure 4B and Table 1).

RLS_40_ tumour tissue was initially characterised by a certain level of spontaneous apoptosis: the volume density of caspase-7 positive cells was 5.3 ± 0.5% of the total tumour tissue (Figure 4C and Table 1) and that of caspase-3 was 2.5 ± 0.3% (Figure S1). The binase administration resulted in apoptosis induction, which was manifested by an increase in the volume density of caspase-7-positive cells by 4.9- and 3.4-fold at the binase doses of 0.5 and 1 mg/kg, respectively, compared with the control group (Figure 4C and Table 2). Caspase-3-positive cells were not detected in the tumour after the binase treatment, which was most likely due to the fact that caspase-3 was not involved in the early events of apoptosis. Additionally, necrotic changes and inflammatory infiltration, represented predominantly by lymphocytes, were found in the RLS_40_ tumours in both the control and the experimental groups (Table 2). Areas of inflammation in the tumour tissue were reduced by 1.4- and 1.3-fold after the binase administration (0.5 and 1 mg/kg, respectively), while areas of necrosis were similar in all groups (Table 2).

### 3.5. Toxicity and Immunomodulatory Effect of Binase in RLS_40_-Bearing Mice

To assess the toxic effects of binase, the biochemical parameters of the blood serum and morphological changes in the liver and kidneys were evaluated for the experimental and control groups. The blood biochemistry of the mice with RLS_40_ showed that tumour development led to 1.7- and 1.2-fold increases in the AST and creatinine levels, respectively, in comparison with the healthy animals (Table S3). The administration of binase at a dose of 0.5 mg/kg did not affect hepatic toxicity or levels of hepatic enzymes, while binase at a dose of 1 mg/kg caused both an increase in hepatic toxicity and levels of ALT, AST and ALK by factors of 1.5, 3.1 and 2, respectively, compared to the healthy animals and by factors of 1.5, 1.9 and 1.7, respectively, compared to the control group (Table S3).

The histological analysis of the liver and kidney tissues revealed that the tumour growth was accompanied by moderate destructive changes in the liver and kidney parenchyma, represented by dystrophy and necrosis of the hepatocytes and epitheliocytes of the proximal tubules (Figure S2). The low doses of binase did not additionally increase the liver and kidney destruction (Figure S2), while binase applied at a dose of 1 mg/kg enhanced the destructive changes, especially in the liver, with a predominance of irreversible necrotic changes (Figure S2). This suggests that the antitumour activity of binase applied at a dose over 1 mg/kg was limited by hepatic toxicity, which was aggravated by tumour metastases in the liver.

The morphometric study of the spleen and thymus revealed that the tumour progression was accompanied by moderate immunomodulatory effects: the volume density of white pulp and the diameter of lymphoid follicles increased by 1.2-fold compared to the healthy animals (Table S2 and Figure S2). The binase administration caused pronounced signs of spleen activation: an increase in the size and number of lymphoid follicles, their fusion and the formation of germinal centres in them (Figure S2 and Table S4). In the thymus of RLS_40_-bearing mice, the volume densities of the cortex and medulla in the experimental and control groups did not differ significantly from these parameters in the healthy animals (Figure S2 and Table S4). Thus, morphofunctional changes in the spleen and thymus indicated non-specific activation of the immune system during binase therapy.

### 3.6. The Effect of Binase on miRNA Profile of the Tumour Tissue and Blood Serum of Mice with RLS_40_

**Figure 5:**
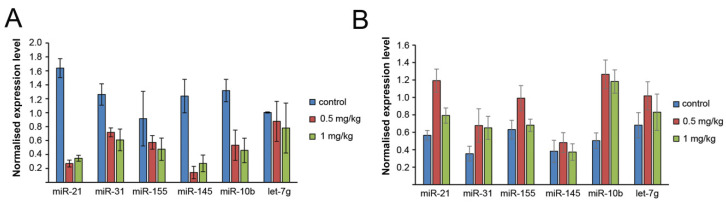
The effect of binase on the level of miRNA in the tumour tissue and bloodstream of mice with RLS_40_. One hour after the last injection of binase, tumour and blood samples were taken, total RNA was isolated and the miRNAs were analysed using RT-qPCR. The miRNA profile in the blood of healthy animals was used for comparison with the miRNA profiles in mice with RLS_40_ treated with saline buffer or with binase. (**A**) Analysis of the miRNA levels in blood serum and the expression level in tumour tissue (**B**) of mice with RLS_40_ after the binase treatment at the doses of 0.5 and 1 mg/kg. The expression level of miRNAs in the tumour tissue was normalised to U6. The concentration of serum-derived miRNAs was normalised to the serum volume. Statistical analysis was performed using one-way ANOVA with the Tukey’s post hoc test; *p* < 0.05 was considered to be statistically significant.

The miRNA profiles in tumour tissue and blood serum of RLS_40_-bearing mice after the binase treatment were analysed using stem-loop RT-qPCR. As we expected, the binase treatment led to a decrease in the levels of five out of six analysed miRNAs in the serum (Figure 5A), excluding let-7g, which remained at the same level as in the control group. An interesting finding was that, in contrast to the fact that in vitro binase caused a reduction in the levels of four out of six analysed miRNAs (Figure 1E), in the tumour tissue, the binase administration caused an increase in the levels of five miRNAs: oncomirs miR-21, miR-10b, miR-31 and miR-155 and the oncosupressor let-7g (Figure 5B). No effect of the binase administration was found on the level of miR-145a.

### 3.7. Pathways Controlled by the Revealed Binase-Susceptible miRNAs

**Figure 6:**
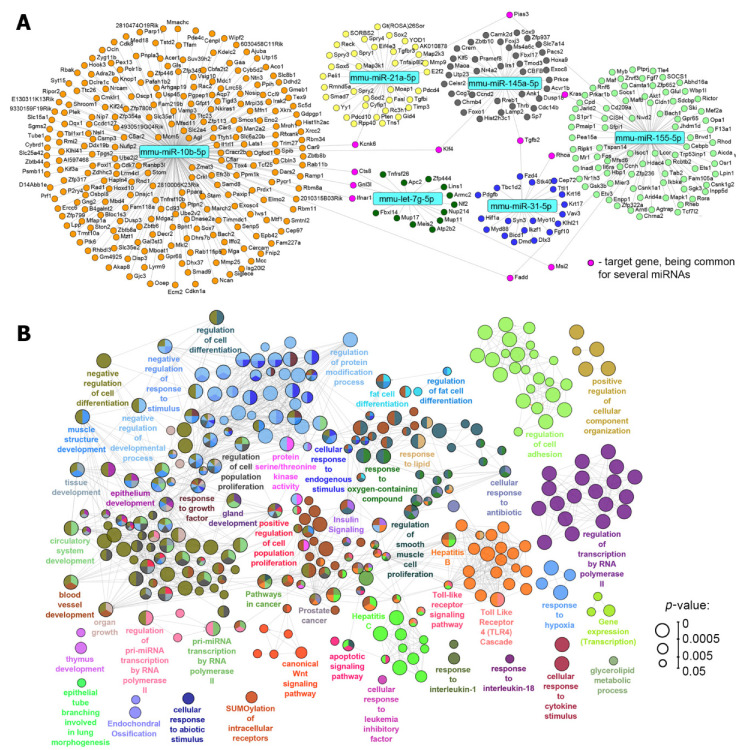
Functional annotation of the binase-susceptible miRNAs identified in RLS_40_ cells. (**A**) Regulome of miRNAs and their target genes reconstructed using the miRTarBase 8.0 (*Mus musculus*) database. The analysis was performed using the CyTargetLinker plugin and the network was visualised using Cytoscape 3.7.2. (**B**) The interaction network of significant terms enriched with the revealed miRNA target genes. Functional annotation was performed on ClueGO by using GeneOntology (biological processes), KEGG, REACTOME and Wikipathways. The functionally grouped network was linked based on the kappa scores of the terms. Only terms/pathways with *p* < 0.05 after the Bonferroni correction were included in the network.

We then questioned which biological processes and pathways could be controlled by the revealed binase-susceptible miRNAs and whether the modulatory effect of binase on these miRNAs could underlie its biological activities, including those reported previously [21,22,23,24,25,26,27,28,32,33]. In order to understand this, we restored all the target genes of the analysed miRNAs miR-21a, miR-145a, miR-31, miR-10b, miR-155 and let-7g using the miRTarBase database, containing only experimentally validated miRNA-target interactions [40]. As depicted in Figure 6A, the reconstructed regulome was characterised by a clustered structure: each distinct miRNA was found to be linked to its own list of target genes and only a limited number of targets was detected as being common to several miRNAs.

When analysing gene targets of miRNA in internal miRNA networks, it was revealed that the oncomirs miR-21a, miR-10b, miR-31 and miR-155 modulated the functions of a great number of genes related to cell transformation, e.g., tumour suppressors, inhibitors of proliferation and migration and mediators of apoptosis (Figure 6A and Tables S5, S6, S8 and S9). It should be noted that the oncosuppressors miR-145a and let-7g have practically no targets related to the enhancement of tumour progression (Tables S7 and S10).

The key common genes with an expression that was modulated by the evaluated miRNAs were analysed from the point of view of their participation in cancer-related pathways and events using the Gene Card and KEGG pathway databases [42,43]. Eleven common genes were found to join the miRNA networks: *CTSB*, *GNL3L*, *IFNAR1*, *KCNK6*, *KIF4A*, *PIAS3*, *KRAS*, *RHOA*, *MSI2*, *FADD* and *TGFB2* (Table 3). Considering the events and pathways in which these genes are involved in tumour progression let us discover that the main modulated pathways included apoptosis and autophagy, proliferation, cancer-related pathways (JAK-STAT, PI3K-Akt, Wnt, MAPK Erk, Ras signalling, oncogenic MAPK signalling, ERK signalling, AGE/RAGE, PKA and TGF-beta pathways; Table 3), inflammation, angiogenesis, adhesion and miRNA in cancer (Table 3).

From the point of view of the housekeeping gene function, very interesting findings were related to rRNA processing, regulation of the expression of mRNAs at the translation level, protein turnover, regulation of the potassium channel activity and transcription-coupled nucleotide excision repair (Table 3). Thus, common genes of the evaluated miRNA networks can be considered as common players in the maintenance of cell integrity, transcription and translation, and the modulation of cancer-related pathways.

In order to deeply understand these intracellular processes that can be regulated by the analysed miRNAs, comprehensive functional annotation of the revealed target genes was performed using the GeneOntology, KEGG, REACTOME and WikiPathways databases. The obtained pathway network showed that alterations in the levels of binase-susceptible miRNAs could affect a wide spectrum of processes and signalling pathways in cells (Figure 6B). It was found that the majority of the revealed terms were associated with processes that have already been identified as susceptible to binase action. The high enrichment of terms related to apoptosis, cell viability and proliferation of tumour cells agreed well with the ability of binase to trigger apoptosis in RLS_40_ cells, as described in this work. Besides this, our data demonstrated the expediency of further investigation of binase as a promising modulator of the tumour microenvironment (Figure 6B): high enrichment of pathways associated with the differentiation of cells (e.g., “regulation of cell differentiation” and “tissue development”) and angiogenesis (“blood vessel development”, “circulatory system development” and “regulation of smooth muscle cell proliferation”).

For the analysis, the following databases were used: GeneCards and KEGG pathways [42,43].

## 4. Discussion

Here, we studied the mechanism of the antitumour activity of binase using mouse lymphosarcoma RLS_40_ focused in three main directions: (1) an investigation into the antitumour activity of binase in vitro in tumour cells; (2) the study of the potential of anticancer therapy with binase using the RLS_40_ tumour model in vivo, paying special attention to the dose dependence of the antitumour effects and binase toxicity; (3) a search for correlations between the cytotoxic (in vitro) and antitumour (in vivo) effects of binase and changes in the miRNA profile, both in tumour cells/tissue and serum caused by the enzyme.

Our data obtained in vitro showed that binase exhibited cytotoxic effects towards RLS_40_ cells via the induction of apoptosis and changes in the level of a number of miRNAs. Binase induced apoptosis through the activation of caspase-3/-7, which was observed for caspase-3/-7 in vitro and for caspase-7 both in vitro and in vivo. This is consistent with previously obtained data that indicated the activation of apoptotic responses by binase in human A549 alveolar adenocarcinoma cells [49], Kasumi-1 acute myeloid leukaemia cells [23] and mouse B16 melanoma cells [28].

The study of binase’s effects on tumour progression in mice revealed that therapy with binase essentially retarded the growth of primary tumours and inhibited metastases development in the liver, thus showing excellent results, especially because binase was used as a monotherapy for a tumour with a multiple-drug-resistant phenotype [28]. Interestingly, binase exhibited a pronounced antitumour effect at doses of 0.5 and 1 mg/kg, both in terms of the primary tumour growth and metastases development. Nevertheless, binase at the dose of 1 mg/kg worsened the blood biochemical parameters, indicating the toxicity of binase in the liver and kidney. However, we did not observe any statistically significant differences regarding the complete blood count (CBC).

We considered the ability of binase to affect miRNAs using a panel of six miRNAs earlier evaluated as a result of an NGS analysis of the miRNA profile in the serum and tumour tissue of mice with Lewis lung carcinoma [46]. As we expected, in the bloodstream of tumour-bearing mice, binase decreased the levels of five analysed miRNAs, except for let-7g. However, regarding the effects of binase on the miRNA levels in tumour cells in vitro and in tumour tissue in vivo, we obtained contradictory results. In in vitro RLS_40_ cells, binase decreased the expression levels of four out of six tested miRNAs, whereas in the tumour tissue, binase caused an increase in the levels of five out of six miRNAs. Such contradictory effects of binase can be explained in several ways. First of all, the dose used in vitro was many times higher than the dose used in vivo: 0.5 mg/mL used in vitro corresponded to the dose of 0.5 g/kg in comparison with the dose of 0.5–1 mg/kg used in vivo. In previous work, we demonstrated the pronounced antitumour and antimetastatic potential of binase for doses of just 0.5–1 mg/kg [28]. Since the ability of binase to penetrate into the cell and nucleus has been shown [49], and its stability in cells for 48 h has been demonstrated [36], we can suggest that the observed decrease in miRNA levels in vitro was due to total RNA degradation, including pre-miRNAs, as it was shown for onconase [50] and mature miRNAs. As for the increase in the expression of intracellular miRNAs in RLS_40_ tumour tissue, this may be associated with the combined systemic effects of binase in vivo. Binase cleaves miRNAs in the bloodstream, leading to a decrease in their levels and a deficiency regarding building a pre-metastatic niche. The observed upregulation of miRNA expression in the tumour tissue may be related to both a deficiency in miRNA in the bloodstream, as well as with the acceleration of miRNA biogenesis in the tumour tissue due to the cleavage of pre-miRNAs, pri-miRNAs and mature miRNAs. Thus, a feedback system was released that replenished the level of miRNAs required for the formation of a pre-metastatic niche and tumour microenvironment. Furthermore, we cannot exclude the fact that since the measurement of the miRNA levels was done at the end of the experiment when the tumour sizes in the experimental group were significantly smaller compared to the control group, clones of cells with an unaltered expression of miRNA were maintained, which were necessary for the tumour to progress, and all other cells died due to RNA degradation and the induction of apoptosis.

Binase’s ability to decrease the miRNA levels in the bloodstream of mice with RLS_40_ was in accordance with data of the decrease of the levels of most miRNAs in the bloodstream of LLC-bearing mice after the treatment with bovine pancreatic RNase A [46]. In the case of tumour cells in vitro and tumour tissue in vivo, it should be noted that the effect of bovine pancreatic RNase A on the miRNA profile of the LLC model differed from the effect of binase on the same miRNAs in the RLS_40_ model. The reasons are: (1) the pyrimidine-X cleavage specificity of RNase A and the guanine-X cleavage specificity of binase; (2) targets of RNase A are extracellular RNAs, whereas targets for binase are both intracellular and extracellular RNAs. Some evidence has been obtained showing that binase cleaves cellular non-coding RNAs [36]. Several studies have demonstrated the ability of onconase and pancreatic RNase A to affect tumour and circulating miRNAs. In a malignant pleural mesothelioma cell line, onconase significantly up-regulated *hsa*-miR-17 and down-regulated *hsa*-miR-30c, which resulted in NF-κB inhibition and an increase in the chemosensitivity of tumour cells [51]. Another in vitro study in the mesothelioma cell line Msto-211h showed that onconase down-regulated intracellular miRNAs via the cleavage of miRNA precursors [50]. Interestingly, RNase A affects the level of miRNA in tumour cells in vitro and in tumour tissue in vivo in a similar way, i.e., the up-regulation of their expression [46]. Since RNase A cannot cleave intracellular RNA due to the binding with a ribonuclease inhibitor, it was suggested that RNase A can play the role of a transcription factor similar to angiogenin [46].

Analysis of the obtained miRNA networks showed that modulation of the expression of binase-susceptible miRNAs can affect a wide spectrum of processes in cells, including events related to cell transformation, proliferation, apoptosis, cancer-related pathways, angiogenesis and adhesion. Other processes found to be triggered by binase were inflammation and some housekeeping functions, such as ribosome biogenesis and transport. Interestingly, the analysis of common genes for external miRNA networks and genes for internal miRNA-networks revealed *Kras*, a verified target of binase [52], and a range of genes with expressions that are sensitive to other RNases, including *Acvr1b*, *Ccnd2* and *Gaa* (sensitive to RNase A) [53] and *p21* (sensitive to RNases L and MC2) [54,55]. Additionally, the significant associations of target genes with inflammation, virus reproduction (“Hepatitis C” and “Hepatitis B”) and cell adhesion were in good agreement with the previously reported activities of binase, including its stimulating effect on M1 macrophage polarisation [56] and the expression of TNF and NF-κB-related genes in leukemic Kasumi-1 cells [23]. Moreover, binase has inhibitory effects on the replication of RNA viruses [30,34] and the motility of tumour cells [57].

## 5. Conclusions

Our findings using a bioinformatics approach not only verified the tight linkage between the effect of binase on the expression levels of selected miRNAs and events mediating cell transformations but also showed that binase-susceptible miRNAs may be involved in the mechanisms underlying the other activities of this enzyme, with an impact on the formation of a pre-metastatic niche and tumour microenvironment. Additionally, binase can probably affect the differentiation of tumour microenvironment-related cells, cancer stem cells and tumour-associated macrophages or fibroblasts, and may inhibit vascularisation of the tumour site. These data provide a reasonable foundation for further investigations into binase as a modulator of antitumour responses during the various steps of tumour progression.

## Figures and Tables

**Table 1:** The levels of miRNAs in RLS_40_ cells and altered levels after the cell treatment with binase in vitro.

Number in the Library According to RPKM *	Top miRNA (LLC NGS Data) [46]	miRNA sequence, 5′→3′	The Number of Guanine Residues ^#^	Expression Level of miRNAs in the RLS_40_ cells ^##^	The Alteration of the miRNA Levels after the Binase Treatment ^##^
1	mir-21a	UA**G**CUUAUCA**G**ACU**G**AU**G**UU**G**A	5	1.7	2.0↓
10	mir-145a	**G**UCCA**G**UUUUCCCA**GG**AAUCCCU	4	1.4	no effect
15	mir-31	A**GG**CAA**G**AU**G**CU**GG**CAUA**G**CUG	7	0.85	1.6↓
29	mir-10b	UACCCU**G**UA**G**AACC**G**AAUUU**G**UG	4	1.5	no effect
46	let-7g	U**G**A**GG**UA**G**UA**G**UUU**G**UACA**G**UU	7	1.8	1.6↓
47	miR-155	UUAAU**G**CUAAUU**G**U**G**AUA**GGGG**U	7	1.9	1.8↓

	U6 snRNA **			0.40	

RLS_40_ cells were treated with binase (5 μg/mL) for 48 h. The absolute values of miRNA in RLS_40_ cells were obtained using qPCR. * RPKM (reads per kilobyte per million): number of reads of specific miRNA/(size of miRNA (kb) × total number of reads in library (million)); ** U6 snRNA was used as an internal control for the miRNA level normalisation; ^#^ guanine residues susceptible to cleavage with binase are indicated; ^##^ miRNA levels normalised to U6 snRNA.

**Table 2:** Morphological organisation of RLS_40_ tumour tissue.

Morphological Parameter	Control	Binase (0.5 mg/kg)	Binase (1 mg/kg)
Unchanged tumour tissue, Vv (%)	71.2 ± 1.3	74.5 ± 1.7 ^#^	74.4 ± 1 ^#^
Lymphoid infiltration, Vv (%)	19.5 ± 1.4	14.3 ± 0.9 ^#^	15 ± 0.9 ^#^
Necrotic changes, Vv (%)	9 ± 0.9	10.9 ± 2	10.3 ± 0.8
Mitotic cells, Nv	5.6 ± 0.4	1.1 ± 0.3 ^#^	3.2 ± 1 ^#^
PCNA positive cells, Vv (%)	62.7 ± 3.8	21.4 ± 2.3 ^#^	44.7 ± 9.5 ^#^
Caspase-7 positive cells, Nv	5.3 ± 0.5	25.9 ± 2.3 ^#^	17.8 ± 3.5 ^#^

Control: RLS_40_-bearing mice treated with a saline buffer. ^#^ Differences from the control were significant at *p* ≤ 0.05.

**Table 3:** Common target genes of the evaluated miRNAs and their functions.

Event	Target Gene	Description	miRNAs	Pathway
Apoptosis	*CTSB*	Cathepsin C	miR-10b/let-7g	Apoptosis and autophagy Apoptosis modulation and signalling Caspase activation via an extrinsic apoptotic signalling pathway
	*KRAS*	KRAS Proto-Oncogene, GTPase	miR-155/miR-145a	Apoptosis pathway
	*FADD*	Fas Associated via Death Domain	miR-155/miR-10b	Apoptosis modulation and signalling Apoptosis and autophagy
	*TGFB2*	Transforming Growth Factor Beta 2	miR-145a/miR-31	Cellular apoptosis pathway Mitochondrial apoptosis
Proliferation	*KIF4A*	Kinesin Family Member 4A	miR-10b/miR-145a	L1CAM interactions
	*TGFB2*	Transforming Growth Factor Beta 2	miR-145a/miR-31	TGF-beta pathway
Cancer-related pathways	*IFNAR1*	Interferon Alpha and Beta Receptor Subunit 1	miR-10b/let-7g	JAK/STAT signalling pathway PI3K/Akt signalling pathway
	*PIAS3*	Protein Inhibitor of Activated STAT 3	miR-155/miR-21a	JAK/STAT signalling pathway
	*KRAS*	KRAS Proto-Oncogene, GTPase	miR-155/miR-145a	MAPK Erk pathway Ras signalling pathway Oncogenic MAPK signalling
	*RHOA*	Ras Homolog Family Member A	miR-155/miR-31	ERK signalling Wnt signalling pathway AGE/RAGE pathway
	*FADD*	Fas Associated Via Death Domain	miR-155/miR-10b	PI3K/Akt signalling pathway
	*KCNK6*	Potassium Two-Pore Domain Channel Subfamily K Member 6	miR10b/miR-21a	PKA signalling
	*TGFB2*	Transforming Growth Factor Beta 2	miR-145a/miR-31	TGF-β pathway
Immunity/ inflammation	*PIAS3*	Protein Inhibitor of Activated STAT 3	miR-155/miR-21a	IL-6-mediated signalling
	*RHOA*	Ras Homolog Family Member A	miR-155/miR-31	CCR5 pathway in macrophages (chemokine signalling)
	*FADD*	Fas Associated via Death Domain	miR-155/miR-10b	Toll-like receptor 4 (TLR4) cascade
	*TGFB2*	Transforming Growth Factor Beta 2	miR-145a/miR-31	Toll-like receptor signalling pathway Plasmin signalling
Angiogenesis	*TGFB2*	Transforming Growth Factor Beta 2	miR-145a/miR-31	Angiogenesis
Adhesion	*RHOA*	Ras Homolog Family Member A	miR-155/miR-31	Cytoskeleton remodelling (cell adhesion and migration) Adherens junction
	*TGFB2*	Transforming Growth Factor Beta 2	miR-145a/miR-31	Cell adhesion
miRNA in cancer	*KRAS*	KRAS Proto-Oncogene, GTPase	miR-155/miR-145a	Silencing of tumour suppressor genes
	*MSI2*	Musashi RNA Binding Protein 2	miR-155/let-7g	mRNA surveillance pathway
	*TGFB2*	Transforming Growth Factor Beta 2	miR-145a/miR-31	MicroRNAs in cancer
House-keeping functions	*GNL3L*	G Protein Nuclear 3 Like	miR-10b/let-7g	Ribosome biogenesis in eukaryotes
	*KCNK6*	Potassium Two-Pore Domain Channel Subfamily K Member 6	miR-10b/miR-21a	Potassium channels Hepatic ABC transporters
	*PIAS3*	Protein Inhibitor of Activated STAT 3	miR-155/miR-21a	Transcription-coupled nucleotide excision repair (TC-NER)

## Supplementary Materials

The following are available online at https://www.mdpi.com/2218-273X/10/11/1509/s1, Figure S1: Caspase-3-positive cells in RLS_40_ tumour tissue after binase administration, Figure S2: Representative histological images of liver, kidney, spleen and thymus sections, Table S1: The primer sequence for the reverse transcription (RT) of miRNAs, Table S2: Sequences of the forward and reverse miRNA-specific primers for qPCR, Table S3: Blood biochemistry of healthy mice and RLS_40_-bearing mice treated with a saline buffer and binase at the doses of 0.5 and 1 mg/kg, Table S4: Morphological parameters of the spleen and thymus of healthy mice and RLS_40_-bearing mice treated with a saline buffer and binase at the doses of 0.5 and 1 mg/kg, Table S5: Gene targets for mmu-miR-21a participating in pro-oncogenic events and pathways, Table S6: Gene targets for mmu-miR-10b participating in pro-oncogenic events and pathways, Table S7: Gene targets for mmu-miR-31 participating in pro-oncogenic events and pathways; Table S8: Gene targets for mmu-miR-145 participating in pro-oncogenic events and pathways, Table S9: Gene targets for mmu-miR-155 participating in pro-oncogenic events and pathways, Table S10: Gene targets for mmu-let-7-g participating in pro-oncogenic events and pathways.

## References

[1] Shi Y., Jin Y. (2009). MicroRNA in cell differentiation and development. Sci. China C Life Sci..

[2] Bartel D.P. (2004). MicroRNAs: Genomics, biogenesis, mechanism, and function. Cell.

[3] Ambros V. (2004). The functions of animal microRNAs. Nature.

[4] Guo H., Ingolia N.T., Weissman J.S., Bartel D.P. (2010). Mammalian microRNAs predominantly act to decrease target mRNA levels. Nature.

[5] Taby R., Issa J.P. (2010). Cancer epigenetics. CA Cancer J. Clin..

[6] Jeyapalan Z., Deng Z., Shatseva T., Fang L., He C., Yang B.B. (2011). Expression of CD44 3′-untranslated region regulates endogenous microRNA functions in tumorigenesis and angiogenesis. Nucleic Acids Res..

[7] Wang J.X., Jiao J.Q., Li Q., Long B., Wang K., Liu J.-P., Li Y.-R., Li P.-F. (2011). miR-499 regulates mitochondrial dynamics by targeting calcineurin and dynamin-related protein-1. Nat. Med..

[8] Pichiorri F., Suh S.S., Rocci A., De Luca L., Taccioli C., Santhanam R., Zhou W., Benson D.M., Hofmainster C., Alder H. (2010). Downregulation of p53-inducible microRNAs 192, 194, and 215 impairs the p53/MDM2 autoregulatory loop in multiple myeloma development. Cancer Cell.

[9] Wu D.W., Cheng Y.W., Wang J., Chen C.Y., Lee H. (2010). Paxillin predicts survival and relapse in non-small cell lung cancer by microRNA-218 targeting. Cancer Res..

[10] Yang F., Xian R.R., Li Y., Polony T.S., Beemon K.L. (2007). Telomerase reverse transcriptase expression elevated by avian leukosis virus integration in B cell lymphomas. Proc. Natl. Acad. Sci. USA.

[11] McCarthy N. (2010). Cancer: Small losses, big gains with microRNAs. Nat. Rev. Genet..

[12] Mironova N., Vlassov V. (2019). Surveillance of tumour development: The relationship between tumour-associated RNAs and ribonucleases. Front. Pharmacol..

[13] Endo Y., Huber P.W., Wool I.G. (1983). The ribonuclease activity of the cytotoxin alpha-sarcin. The characteristics of the enzymatic activity of alpha-sarcin with ribosomes and ribonucleic acids as substrates. J. Biol. Chem..

[14] Kao R., Shea J.E., Davies J., Holden D.W. (1998). Probing the active site of mitogillin, a fungal ribotoxin. Mol. Microbiol..

[15] Lacadena J., Alvarez-Garcia E., Carreras-Sangra N., Herrero-Galan E., Alegre-Cebollada J., Garcia-Ortega L., Oñaderra M., Gavilanes J.G., Martínez del Pozo A. (2007). Fungal ribotoxins: Molecular dissection of a family of natural killers. FEMS Microbiol. Rev..

[16] Pouckova P., Zadinova M., Hlouskova D., Strohalm J., Plocová D., Spunda M., Olejár T., Zitko M., Matousek J., Ulbrich K. (2004). Polymer-conjugated bovine pancreatic and seminal ribonucleases inhibit growth of human tumors in nude mice. J. Control. Release.

[17] Lee I., Lee Y.H., Mikulski S.M., Lee J., Covone K., Shogen K. (2000). Tumoricidal effects of onconase on various tumors. J. Surg. Oncol..

[18] Patutina O.A., Mironova N.L., Ryabchikova E.I., Popova N.A., Nikolin V.P., Kaledin V.I., Vlassov V.V., Zenkova M.A. (2010). Tumoricidal activity of RNase A and DNase I. Acta Naturae.

[19] Patutina O., Mironova N., Ryabchikova E., Popova N., Nikolin V., Kaledin V., Vlassov V.V., Zenkova M.A. (2011). Inhibition of metastasis development by daily administration of ultralow doses of RNase A and DNase I. Biochimie.

[20] Prior T., Kunwar S., Pastan I. (1996). Studies on the activity of barnase toxins in vitro and in vivo. Bioconjugate Chem..

[21] Makarov A.A., Ilinskaya O.N. (2003). Cytotoxic ribonucleases: Molecular weapons and their targets. FEBS Lett..

[22] Giancola C., Ercole C., Fotticchia I., Spadaccini R., Pizzo E., D’Alessio G., Picone D. (2011). Structure-cytotoxicity relationships in bovine seminal ribonuclease: New insights from heat and chemical denaturation studies on variants. FEBS J..

[23] Mitkevich V.A., Kretova O.V., Petrushanko I.Y., Burnysheva K.M., Sosin D.V., Simonenko O.V., Ilinskaya O.N., Tchurikov N.A., Makarov A.A. (2013). Ribonuclease binase apoptotic signature in leukemic Kasumi-1 cells. Biochimie.

[24] Makarov A.A., Kolchinsky A., Ilinskaya O.N. (2008). Binase and other microbial RNases as potential anticancer agents. BioEssays.

[25] Ardelt W., Ardelt B., Darzynkiewicz Z. (2009). Ribonucleases as potential modalities in anticancer therapy. Eur. J. Pharmacol..

[26] Fang E.F., Ng T.B. (2011). Ribonucleases of different origins with a wide spectrum of medicinal applications. Biochim. Biophys. Acta.

[27] Ulyanova V., Vershinina V., Ilinskaya O. (2011). Barnase and binase: Twins with distinct fates. FEBS J..

[28] Mironova N.L., Petrushanko I.Y., Patutina O.A., Sen’kova A.V., Simonenko O.V., Mitkevich V.A., Markov O.V., Zenkova M.A., Makarov A.A. (2013). Ribonuclease binase inhibits primary tumor growth and metastases via apoptosis induction in tumor cells. Cell Cycle.

[29] Garipov A.R., Nesmelov A.A., Cabrera-Fuentes H.A., Ilinskaya O.N. (2014). Bacillus intermedius ribonuclease (BINASE) induces apoptosis in human ovarian cancer cells. Toxicon.

[30] Shah Mahmud R., Ilinskaya O.N. (2013). Antiviral activity of binase against the pandemic influenza A (H1N1) virus. Acta Naturae.

[31] Shah Mahmud R., Mostafa A., Müller C., Kanrai P., Ulyanova V., Sokurenko Y., Dzieciolowski J., Kuznetsova I., Ilinskaya O., Pleschka S. (2018). Bacterial ribonuclease binase exerts an intra-cellular anti-viral mode of action targeting viral RNAs in influenza a virus-infected MDCK-II cells. Virol. J..

[32] Kurinenko B.M., Sobchuk L.I., Khaĭbullina S.A., Bulgakova R.S. (1988). Experimental research on the antitumor effectiveness of Bac. intermedius RNAse. Eksp Onkol..

[33] Kurinenko B.M., Sergeeva E.V., Sobchuk L.I., Bulgakova R.S., Khaĭbullina S.A. (1989). In vitro and in vivo studies of RNAse of Bacillus intermedius. Antibiot Khimioter.

[34] Mitkevich V.A., Petrushanko I.Y., Spirin P.V., Fedorova T.V., Kretova O.V., Tchurikov N.A., Prassolov V.S., Ilinskaya O.N., Makarov A.A. (2011). Sensitivity of acute myeloid leukemia Kasumi-1 cells to binase toxic action depends on the expression of KIT and AML1-ETO oncogenes. Cell Cycle.

[35] Mit’kevich V.A., Orlova N.N., Petrushanko I., Simonenko O.V., Spirin P.V., Prokofeva M.M., Gornostaeva A.S., Stocking C., Makarov A.A., Prasolov V.S. (2013). Expression of FLT3-ITD oncogene confers mice progenitor B-cells BAF3 sensitivity to the ribonuclease binase cytotoxic action. Mol. Biol..

[36] Mitkevich V.A., Tchurikov N.A., Zelenikhin P.V., Petrushanko I.Y., Makarov A.A., Ilinskaya O.N. (2010). Binase cleaves cellular noncoding RNAs and affects coding mRNAs. FEBS J..

[37] Chen C., Ridzon D.A., Broomer A.J., Zhou Z., Lee D.H., Nguyen J.T., Barbisin M., Xu N.L., Mahuvakar V.R., Andersen M.R. (2005). Real-time quantification of microRNAs by stem-loop RT-PCR. Nucleic Acids Res..

[38] Varkonyi-Gasic E., Wu R., Wood M., Walton E.F., Hellens R.P. (2007). Protocol: A highly sensitive RT-PCR method for detection and quantification of microRNAs. Plant Methods.

[39] Kutmon M., Kelder T., Mandaviya P., Evelo C.T.A., Coort S.L. (2013). CyTargetLinker: A cytoscape app to integrate regulatory interactions in network analysis. PLoS ONE.

[40] Huang H.Y., Lin Y.C.D., Li J., Huang K.Y., Shrestha S., Hong H.C., Tang Y., Chen Y.G., Jin C.N., Yu Y. (2020). MiRTarBase 2020: Updates to the experimentally validated microRNA-target interaction database. Nucleic Acids Res..

[41] Bindea G., Mlecnik B., Hackl H., Charoentong P., Tosolini M., Kirilovsky A., Fridman W.H., Pagès F., Trajanoski Z., Galon J. (2009). ClueGO: A cytoscape plug-in to decipher functionally grouped gene ontology and pathway annotation networks. Bioinformatics.

[42] Genecards® The Human Gene Database. https://www.genecards.org.

[43] KEGG Kyoto Encyclopedia of Genes and Genomes. https://www.kegg.jp/.

[44] Nakanishi H., Takenaga K., Oguri K., Yoshida A., Okayama M. (1992). Morphological characteristics of tumours formed by Lewis lung carcinoma-derived cloned cell lines with different metastatic potentials: Structural differences in their basement membranes formed in vivo. Virchows Arch. A Pathol. Anat. Histopathol..

[45] Bobek V., Kolostova K., Pinterova D., Kacprzak G., Adamiak J., Kolodziej J., Boubelik M., Kubecova M., Hoffman R.M. (2010). A clinically relevant, syngeneic model of spontaneous, highly metastatic B16 mouse melanoma. Anticancer Res..

[46] Mironova N., Patutina O., Brenner E., Kurilshikov A., Vlassov V., Zenkova M. (2013). MicroRNA drop in the bloodstream and microRNA boost in the tumour caused by treatment with ribonuclease A leads to an attenuation of tumour malignancy. PLoS ONE.

[47] Medina P.P., Nolde M., Slack F.J. (2010). OncomiR addiction in an in vivo model of microRNA-21-induced pre-B-cell lymphoma. Nature.

[48] Eis P.S., Tam W., Sun L., Chadburn A., Li Z., Gomez M.F., Lund E., Dahlberg J.E. (2005). Accumulation of miR-155 and BIC RNA in human B cell lymphomas. Proc. Natl. Acad. Sci. USA.

[49] Cabrera-Fuentes H.A., Aslam M., Saffarzadeh M., Kolpakov A., Zelenikhin P., Preissner K.T., Ilinskaya O.N. (2013). Internalization of Bacillus intermedius ribonuclease (BINASE) induces human alveolar adenocarcinoma cell death. Toxicon.

[50] Qiao M., Zu L.D., He X.H., Shen R.L., Wang Q.C., Liu M.F. (2012). Onconase downregulates microRNA expression through targeting microRNA precursors. Cell. Res..

[51] Goparaju C.M., Blasberg J.D., Volinia S., Palatini J., Ivanov S., Donington J.S., Croce C., Carbone M., Yang H., Pass H.I. (2011). Onconase mediated NFKb downregulation in malignant pleural mesothelioma. Oncogene.

[52] Ilinskaya O.N., Singh I., Dudkina E., Ulyanova V., Kayumov A., Barreto G. (2016). Direct inhibition of oncogenic KRAS by Bacillus pumilus ribonuclease (binase). Biochim. Biophys. Acta-Mol. Cell Res..

[53] Mironova N., Patutina O., Brenner E., Kurilshikov A., Vlassov V., Zenkova M. (2017). The systemic tumor response to RNase A treatment affects the expression of genes involved in maintaining cell malignancy. Oncotarget.

[54] Al-Haj L., Blackshear P.J., Khabar K.S.A. (2012). Regulation of p21/CIP1/WAF-1 mediated cell-cycle arrest by RNase L and tristetraprolin, and involvement of AU-rich elements. Nucleic Acids Res..

[55] Fang E.F., Zhang C.Z.Y., Zhang L., Fong W.P., Ng T.B. (2012). In vitro and in vivo anticarcinogenic effects of RNase MC2, a ribonuclease isolated from dietary bitter gourd, toward human liver cancer cells. Int. J. Biochem. Cell Biol..

[56] Makeeva A., Rodriguez-Montesinos J., Zelenikhin P., Nesmelov A., Preissner K.T., Cabrera-Fuentes H.A., Ilinskaya O.N. (2017). Antitumor macrophage response to bacillus pumilus ribonuclease (Binase). Mediators Inflamm..

[57] Zelenikhin P.V., Ead Mohamed I.S., Nadyrova A.I., Sirotkina A.A., Ulyanova V.V., Mironova N.L., Mit’kevich V.A., Makarov A.A., Zenkova M.A., Ilinskaya O.N. (2020). *Bacillus pumilus* ribonuclease inhibits migration of human duodenum adenocarcinoma HuTu 80 cells. Mol. Biology (Moskow).

