# Supplementary material for: Antitumour Activity of the Ribonuclease Binase from Bacillus pumilus in the RLS40 Tumour Model Is Associated with the Reorganisation of the miRNA Network and Reversion of Cancer-Related Cascades to Normal Functioning
Source: Biomolecules. 2020 Nov 2;10(11):1509. doi: 10.3390/biom10111509 (PMC7692507; doi:10.3390/biom10111509)
Supplement: Supplementary file 1 [file biomolecules-10-01509-s001.pdf]

Supplementary Materials

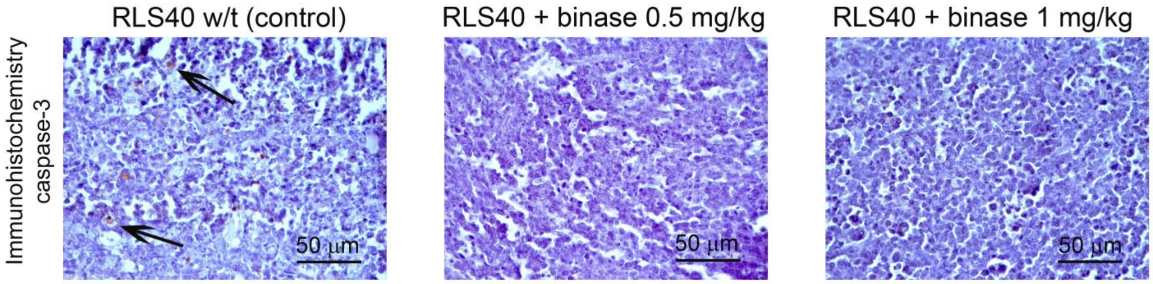

Figure S1. Caspase-3-positive cells in RLS_{40} tumor tissues after binase administration. Representative histological images of tumor sections. Immunohistochemical staining with anti-caspase-3 monoclonal antibodies. Caspase-3-positive cells are indicated by black arrows. Magnification: ×400. Scale bar corresponds to 50 µm.

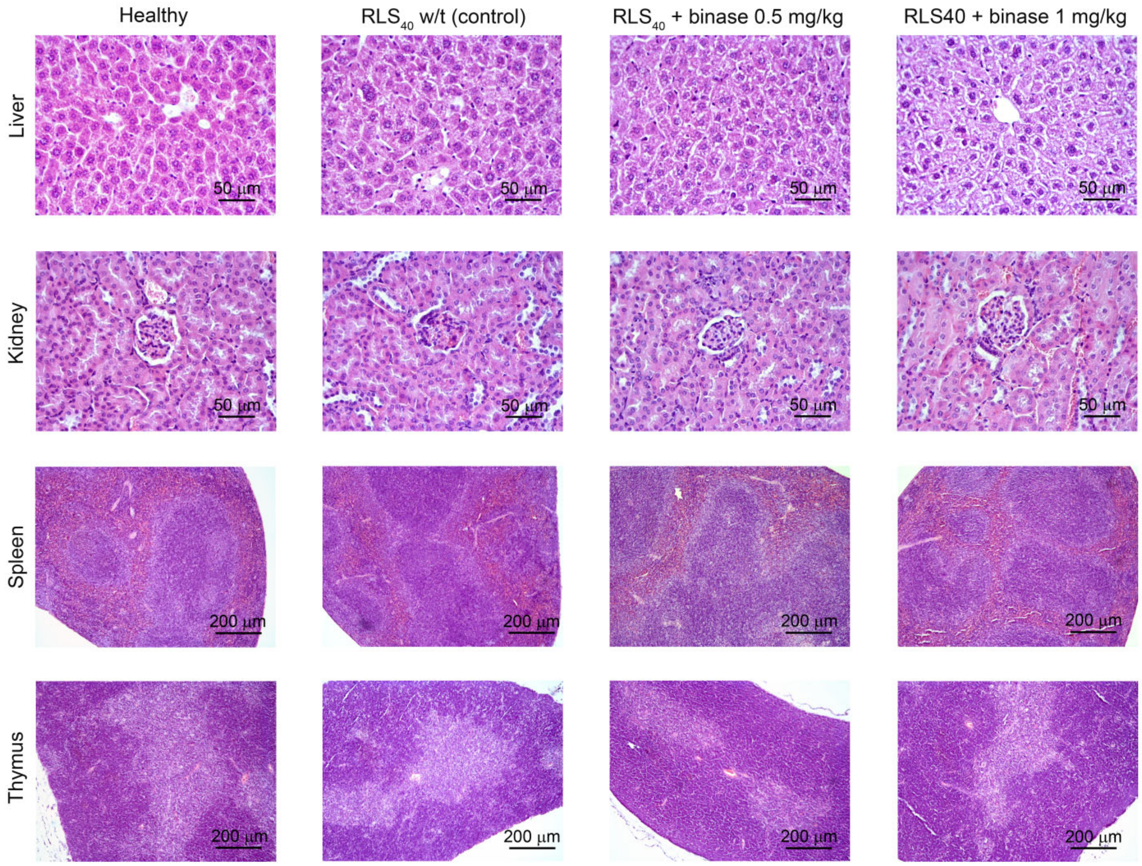

Figure S2. Representative histological images of liver, kidney, spleen and thymus sections. Hematoxylin and eosin staining. Magnification: ×400. Bar corresponds to 50 µm (liver and kidney) and 200 µm (spleen and thymus).

Table S1. The primer sequence for the reverse transcription (RT) of miRNAs.

**Name**	**Sequences of the RT primer, 5' → 3'**
RT mir-31	GTCGTATCCAGTGCAGGGTCCGAGGTATTCGCACTGGATACGACCAGCTATGCC
RT mir-21	GTCGTATCCAGTGCAGGGTCCGAGGTATTCGCACTGGATACGACTCAACATCAG
RT mir-145a	GTCGTATCCAGTGCAGGGTCCGAGGTATTCGCACTGGATACGACAGGGATTCCT
RT mir-155	GTCGTATCCAGTGCAGGGTCCGAGGTATTCGCACTGGATACGACTGGATACGAC
RT mir-10b	GTCGTATCCAGTGCAGGGTCCGAGGTATTCGCACTGGATACGACCACAAATTCG
RT let7-g	GTCGTATCCAGTGCAGGGTCCGAGGTATTCGCACTGGATACGACAACTGTACAA
RT U6	GTCGTATCCAGTGCAGGGTCCGAGGTATTCGCACTGGATACGACAAAAATATGGAACG

Table S2. Sequences of the forward and reverse miRNA-specific primers for qPCR.

**Name**	**Sequences of the Specific Primer, 5’ → 3’**
mir-31-F	AGGCAAGATGCTGGCA
mir-21-F	AGACTAGCTTATCAGACTGA
mir-145a-F	AGGTCCAGTTTTCCCAGGA
mir-155-F	ACTTAATGCTAATTGTGATAGG
mir-10b-F	TACCCTGTAGAACCGAA
let7-g-F	AACGCTGAGGTAGTAGTTTGT
U6-F	CTCGCTTCGGCAGCACA
Universal-R	GTGCAGGGTCCGAGGT

Table S3. Blood biochemistry of healthy mice and RLS_{40}-bearing mice treated with a saline buffer and binase at the doses of 0.5 and 1 mg/kg.

Groups	Biochemical Parameters					
	Liver				Kidneys	
	ALT (U/L)	AST (U/L)	ALK (U/L)	Total Protein (g/L)	Creatinin (mmol/L)	BUN (mmol/L)
Healthy	42 ± 1.6	67.3 ± 1.5	68.1 ± 5.5	55.4 ± 2.1	32.9 ± 1.3	9.9 ± 0.1
Control	39.4 ± 3.4	114.2 ± 13 *	79.8 ± 20	53.4 ± 2.3	39.3 ± 1.3 *	8.4 ± 0.2 *
Binase 0.5 mg/kg	35.6 ± 1.7 *	116.5 ± 14.4 *	130.5 ± 13.5*#	62.4 ± 2.1 *#	31.2 ± 5.1 #	8.8 ± 0.5 *
Binase 1 mg/kg	60.9 ± 10.3 *#	211.4 ± 49.8 *#	134.5 ± 14.8 *#	61.9 ± 3.2 *#	39.2 ± 1.2*	9.7 ± 1.2

Control – RLS_{40}-bearing mice treated with saline buffer.
*The differences from the healthy mice were significant at $p \leq 0.05$.
#The differences from the control mice were significant at $p \leq 0.05$.
ALT: alanine aminotransferase, AST: aspartate aminotransferase, ALK: alkaline phosphatase, BUN: blood urea nitrogen.

Table S4. Morphological parameters of the spleen and thymus of healthy mice and RLS_{40}-bearing mice treated with a saline buffer and binase at the doses of 0.5 and 1 mg/kg.

**Morphological Parameters**	**Healthy**	**Control**	**Binase 0.5 mg/kg**	**Binase 1 mg/kg**
**Spleen**				
Red pulp, Vv (%)	57.3 ± 3.1	49.4 ± 2.9*	31.2 ± 1.1*#	30.4 ± 0.8*#
White pulp, Vv (%)	42.6 ± 1.9	50.4 ± 2.9*	68.5 ± 1.1*#	69.5 ± 0.8*#
Diameter of follicles (µm)	229.9 ± 13.5	270.2 ± 10.1*	322.3 ± 7.6*#	325 ± 11.8*#
**Thymus**				
Cortex, Vv (%)	39.9 ± 1.6	35.1 ± 3.8	40.5 ± 1.1	40.4 ± 2.2
Medulla, Vv (%)	55 ± 1.9	64.8 ± 3.8	59.5 ± 1.1	59.5 ± 2.2
Cortex/medulla index	0.7 ± 0.1	0.7 ± 0.2	0.7 ± 0.03	0.7 ± 0.08

Control – RLS_{40}-bearing mice treated with a saline buffer.
*The differences from the healthy mice were significant at $p \leq 0.05$.
#The differences from the control mice were significant at $p \leq 0.05$.

Table S5. Gene targets for mmu-miR-21a participating in pro-oncogenic events and pathways.

	Target Gene	Description	Signaling Pathway or Event	Function
1	*PDCD10*	Programmed Cell Death 10	- Validated targets of C-MYC transcriptional activation	- Promotes cell proliferation<br>- Modulates apoptotic pathways<br>- Modulates cell migration<br>- Modulates angiogenesis, vasculogenesis and hematopoiesis
2	*PTEN*	Phosphatase and Tensin Homolog	- IL-2 pathway<br>- PI3K/Akt signaling pathway<br>- p53 signaling<br>- MicroRNAs in cancer<br>- T-cell receptor signaling pathway<br>- Pathways in cancer<br>- B-Cell receptor signaling pathway<br>- MAPK signaling;<br>- RhoA signaling pathway	- Tumor suppressor; acts as a dual-specificity protein phosphatase
3	*RC3H1*	Ring Finger and CCCH-Type Domains 1	- STRING interaction network	- Regulation microRNA homeostasis<br>- Post-transcriptional repressor of mRNAs
4	*PDCD4*	Programmed Cell Death 4	- Apoptosis and autophagy<br>- MAPK signaling	- Inhibitor of translation initiation and cap-dependent translation<br>- Inhibits of events important in driving invasion, and consequent JUN-dependent transcription<br>- Plays a role in apoptosis<br>- Inhibits tumor promoter-induced neoplastic transformation
5	*TGFBI*	Transforming Growth Factor Beta Induced	- Adhesion	- Cell adhesion.
6	*TIMP3*	TIMP Metallopeptidase Inhibitor 3	- Matrix metallo-proteinases<br>- Angiogenesis<br>- Cell adhesion, ECM remodeling<br>- MicroRNAs in cancer<br>-VEGF signaling	- Irreversibly inactivates metalloproteinases (MMP-1, MMP-2, MMP-3, MMP-7, MMP-9, MMP-13, MMP-14 and MMP-15).
7	*MMP9*	Matrix Metallopeptidase 9	- Transcriptional misregulation in cancer<br>- Regulation of Wnt-mediated beta catenin signaling and target gene transcription	- Plays an essential role in local proteolysis of the extracellular matrix and in leukocyte migration<br>- Exhibits identical protein binding and metallopeptidase activity

Table S6. Gene targets for mmu-miR-10b participating in pro-oncogenic events and pathways.

	Target Gene	Description	Signaling Pathway or Event	Function
1	*RAB11B*	RAB11B, Member RAS Oncogene Family	- Vesicle-mediated transport<br>- Autophagy pathway<br>- Sertoli–Sertoli cell junction dynamics<br>- Remodeling of the adherens junctions	- Key regulator of intracellular membrane trafficking
2	*MCC*	MCC Regulator of WNT Signaling Pathway	- Wnt pathway	- Suppresses cell proliferation and the Wnt/b-catenin pathway in colorectal cancer cells<br>- Involved in cell migration
3	*VAMP3*	Vesicle Associated Membrane Protein 3	- Vesicle-mediated transport<br>- Sertoli–Sertoli cell junction dynamics	- Involved in vesicular transport from the late endosomes to the trans-Golgi network
4	*LATS1*	Large Tumor Suppressor Kinase 1	- DNA damage<br>- Wnt/Hedgehog/Notch	- Plays a pivotal role in organ size control and tumor suppression by restricting proliferation and promoting apoptosis<br>- Regulates cellular genes important for cell proliferation, cell death, and cell migration<br>- Involves in the control of p53 expression
5	*TCF25*	Transcription Factor 25	- Non-canonical Wnt pathway<br>- Regulation of activated PAK-2p34 by proteasome-mediated degradation	- Play a role in cell death control<br>- Acts as a transcriptional repressor
6	*PTPN2*	Protein Tyrosine Phosphatase Non-Receptor Type 2	- JAK/STAT signaling pathway	- Negatively regulates numerous signaling pathways and biological processes like hematopoiesis, inflammatory response, cell proliferation and differentiation and glucose homeostasis
7	*CDK7*	Cyclin Dependent Kinase 7	- Cyclins and cell cycle regulation<br>- Mitotic G1-G1/S phases<br>- Cell cycle, mitotic<br>- Cell cycle control of chromosomal replication	- Involves in cell cycle control and in RNA-polymerase-II-mediated RNA transcription.
8	*PTK6*	Protein Tyrosine Kinase 6	- Cell cycle, mitotic<br>- Mitotic G1-G1/S phases<br>- Signaling by PTK6	- Implicates in the regulation of a variety of signaling pathways that control the differentiation and maintenance of normal epithelia, as well as tumor growth<br>- Promotes proliferation, migration and invasion.<br>- In cytoplasmic form, it might activate oncogenic signaling pathways
9	*CDKN1A*	Cyclin Dependent Kinase Inhibitor 1A	- Cyclins and cell cycle regulation<br>- p53 signaling	- Involved in p53/TP53-mediated inhibition of cellular proliferation in response to DNA damage

			- Cell cycle, mitotic<br>- JAK/STAT signaling pathway<br>- Apoptosis modulation and signaling<br>- Cell cycle checkpoints	- Plays an important role in controlling cell cycle progression and DNA damage-induced G2 arrest
10	*PRF1*	Perforin 1	- Apoptosis modulation and signaling<br>- Apoptosis and survival caspase cascade	- Plays a key role in secretory granule-dependent cell death<br>- Promotes cytolysis and apoptosis of target cells
11	*DNASE2*	Deoxyribonuclease 2, Lysosomal	- Vesicle-mediated transport<br>- Lysosome	- Plays a major role in the degradation of nuclear DNA in cellular apoptosis during development
12	*PSMB11*	Proteasome Subunit Beta 11	- Cell cycle, mitotic<br>- Cell cycle checkpoints<br>- Mitotic metaphase and anaphase<br>- Apoptotic execution phase	- Plays a pivotal role in the development of CD8-positive T cells<br>- Cleaves peptides with Arg, Phe, Tyr, Leu and Glu

Table S7. Gene targets for mmu-miR-31 participating in pro-oncogenic events and pathways.

	**Target Gene**	**Description**	**Signaling Pathway or Event**	**Function**
1	*PDGFB*	Platelet-Derived Growth Factor Subunit B	- MAPK signaling pathway<br>- Ras signaling pathway<br>- JAK/STAT signaling pathway<br>- MicroRNAs in cancer<br>- Pathways in cancer<br>- PI3K/Akt signaling pathway	- Growth factor that plays an essential role in the regulation of embryonic development, cell proliferation, cell migration, survival and chemotaxis<br>- Plays an important role in wound healing
2	*TBC1D2*	TBC1 Domain Family Member 2	- Vesicle-mediated transport<br>TBC/RABGAPs	- Signal effector participating in the inhibition of cadherin degradation and reduced cell–cell adhesion
3	*FGF10*	Fibroblast Growth Factor 10	- Ras signaling pathway<br>- MAPK signaling pathway<br>- PI3K/Akt signaling pathway<br>- Pathways in cancer	- Plays an important role in the regulation of embryonic development, cell proliferation and cell differentiation

Table S8. Gene targets for mmu-miR-145 participating in pro-oncogenic events and pathways.

	Target Gene	Description	Signaling Pathway or Event	Function
1	*ACVR1B*	Activin A Receptor Type 1B	- Apoptosis pathway<br>- MAPK signaling pathway	- Regulates pathological processes including wound healing, extracellular matrix production, immunosuppression and carcinogenesis
2	*CDC14B*	Cell Division Cycle 14B	- Cell cycle	- Dual-specificity phosphatase involved in the DNA damage response<br>- Important regulator of the G2 DNA damage checkpoint following DNA damage

Table S9. Genes-targets for mmu-miR-155 participating in pro-oncogenic events and pathways.

	Target Gene	Description	Signaling Pathway or Event	Function
1	*CISH*	Cytokine-Inducible SH2-Containing protein	- IL-2 signaling pathway<br>- JAK/STAT signaling pathway<br>- Toll-like receptor signaling pathway	- Involved in the negative regulation of cytokines that signal through the JAK/STAT5 pathway
2	*RHOD*	Ras Homolog Family Member D	- IL-2 pathway<br>- Mitotic prometaphase<br>- Apoptotic pathways in synovial fibroblasts<br>- Molecular mechanisms of cancer<br>- Ras pathway	- May coordinate membrane transport with the function of the cytoskeleton<br>- Modulates the reorganisation of the actin cytoskeleton and focal adhesion dissolution
3	*TSPAN14*	Tetraspanin 14	- Innate immune system	- Regulates the maturation and trafficking of the transmembrane metalloprotease ADAM10<br>- Negatively regulates ADAM10-mediated cleavage of GP6<br>- Promotes the ADAM10-mediated cleavage of CDH5
4	*CSNK1A1*	Casein Kinase 1 Alpha 1	- Wnt signaling pathway<br>- PI3K/Akt signaling pathway<br>- p53 signaling	- Regulates epithelial cell migration<br>- Plays a role in segregating chromosomes during mitosis<br>- Participates in Wnt signaling;<br>- Phosphorylates a large number of proteins
5	*TAB2*	TGF-β-Activated Kinase 1 (MAP3K7) Binding Protein 2	- MAP kinase signaling<br>- IL-1 signaling pathway<br>- Death receptor signaling<br>- Molecular mechanisms of cancer	- Promotes MAP3K7 activation in the IL-1 signaling pathway
6	*PATJ*	PATJ Crumbs Cell Polarity Complex Component	- Cell junction organization<br>- PI3K/Akt signaling pathway<br>- Tight junction	- Regulates protein targeting, cell polarity and the integrity of tight junctions
7	*AGTRAP*	Angiotensin II Receptor Associated Protein	- Oncogenic MAPK signaling	- Negative regulator of type-1 angiotensin II receptor-mediated signaling, as well as a receptor-desensitisation mechanism, such as phosphorylation<br>- Induces a decrease in cell proliferation and angiotensin II-stimulated transcriptional activity
8	*ETV3*	ETS Variant Transcription Factor 3	- Macrophage differentiation and growth	- Transcriptional repressor that contributes to growth arrest during terminal macrophage differentiation<br>- Represses MMP1 promoter activity
9	*TP53INP1*	Tumor Protein P53 Inducible Nuclear Protein 1	- Regulation of TP53 activity mediating the transcription of cell death genes	- Antiproliferative and proapoptotic protein involved in the cell stress response, which acts as a dual regulator of transcription and autophagy

				- Possesses both a p53/TP53-independent intracellular reactive oxygen species (ROS) regulatory function and a p53/TP53-dependent transcription regulatory function<br>- Acts as a tumor suppressor by inducing cell death by an autophagy and caspase-dependent mechanism.
10	*RIPK1*	Receptor-Interacting Serine/Threonine Kinase 1	- Caspase activation via extrinsic apoptotic signalling pathway<br>- Apoptosis modulation and signaling<br>- Apoptosis and Autophagy	- Key regulator of TNF-mediated apoptosis, necroptosis and inflammatory pathways
11	*S1PR1*	Sphingosine-1-Phosphate Receptor 1	- Sphingolipid signaling pathway<br>- IL-4 and IL-13 signaling	- Plays an important role in cell migration<br>- Plays an important role in the regulation of sprouting angiogenesis and vascular maturation
12	*PMAIP1*	Phorbol-12-Myristate-13-Acetate-Induced Protein 1	- Apoptosis modulation and signaling<br>- Apoptosis and autophagy<br>- Pathways in cancer<br>- Molecular mechanisms of cancer<br>- TP53 network	- Promotes the activation of caspases and apoptosis; promotes mitochondrial membrane changes and the efflux of apoptogenic proteins from the mitochondria
13	*SGK3*	Serum/Glucocorticoid Regulated Kinase Family Member 3	- PI3K/Akt signaling	- Involved in the regulation of a wide variety of ion channels, membrane transporters, cell growth, proliferation, survival and migration
14	*GPR65*	G Protein-Coupled Receptor 65	- Gastrin-CREB signaling pathway via PKC and MAPK	- Plays a role in activation-induced cell death or differentiation of T-cells
15	*FGF7*	Fibroblast Growth Factor 7	- Akt signaling<br>- Ras signaling pathway<br>- MAPK signaling pathway<br>- Pathways in cancer	- Plays an important role in the regulation of embryonic development, cell proliferation and cell differentiation
16	*MYB*	MYB Proto-Oncogene, Transcription Factor	- PI3K/Akt signaling pathway<br>- IL-4-mediated signaling events	- Plays an important role in the control of proliferation and differentiation of hematopoietic progenitor cells

Table S10. Gene targets for mmu-let-7-g participating in pro-oncogenic events and pathways.

	Target Gene	Description	Signaling Pathway or Event	Function
1	*APC2*	APC Regulator of WNT Signaling Pathway 2	- PI3K/Akt signaling pathway<br>- MicroRNAs in cancer<br>- Wnt signaling pathway<br>- Pathways in cancer	- May function in Wnt signaling by promoting the rapid degradation of CTNNB1
